# HBx Gene Mutations in Hepatitis B Virus and Hepatocellular Carcinoma

**DOI:** 10.14740/gr589w

**Published:** 2014-03-14

**Authors:** Anumol Mathew M, Sumitha C Kurian, Atul Philip Varghese, Seema Oommen, Manoj G

**Affiliations:** a Corporate R&D Centre, HLL Lifecare Limited, Akkulam, Sreekariyam (PO), Trivandrum 695017, Kerala, India; b Pushpagiri Institute of Medical Science and Research Centre, Thiruvalla, Pathanamthitta, Kerala, India

**Keywords:** HBV, Hepatocellular carcinoma, Mutation, HBx gene

## Abstract

Hepatocellular carcinoma (HCC) is one of the most prevalent cancers which are found in many Asian and African countries. There are several risk factors that may develop to HCC. Along with several other factors contributing to HCC, hepatitis B virus (HBV) infection also accounts for a major cause. HBV infection represents a major health problem worldwide. Among all of HBV genes, HBx is believed to play a prominent role in carcinogenesis, although the actual mechanism is not yet fully understood. The HBx gene of HBV is the most common open reading frame that may undergo mutations and may develop into HCC. This review summarizes the current knowledge about the most important roles of HBx gene that may lead to the development of HCC.

## Introduction

Hepatitis B, a liver disease caused by hepatitis B virus (HBV), is a major health problem across worldwide. WHO estimates that (fact sheet No. 204, updated July 2013) about two billion people have been infected with the HBV whereas more than 240 million are suffering from chronic liver infections. About 600,000 people die every year from the consequences due to hepatitis B infection [1]. Depending on the geographical prevalence of hepatitis B carrier state in general population, countries are categorized into high (8% or more), intermediate (2-7%) or low (< 2%) HBV endemicity. India belongs to intermediate range of HBV endemicity with an estimated total of over 40 million carriers [2]. Many persons with chronic hepatitis may develop hepatocellular carcinoma (HCC). About 50-60% HCC is due to HBV infection [3]. HCC is the fifth most common cancer in men and the nineth in women and is the second most common cause of death from cancer worldwide [4]. The prevalence of HCC is high in Eastern and South-Eastern Asia, Middle and Western Africa as compared with other regions like Europe, North and South America [5, 6].

HBV which belongs to the *hepadnaviridae* family is a small enveloped virus of about 42 nm in diameter. Hepadnaviruses are those that can cause liver infection in humans and animals [7]. The natural host of HBV is humans, whereas the other host range includes apes, woodchucks, squirrels, ducks and cranes [8]. HBV virions contain an outer lipoprotein envelope and a nucleocapsid core within the envelope. The core consists of partially double stranded relaxed circular DNA of about 3.2 kb in size and a polymerase protein that can synthesize viral DNA in infected cells. There are four partially open reading frames (ORFs) which encode the polymerase protein (P gene), core antigen (C gene), surface antigens (Pre S/S) and X proteins (X gene) [9-11].

The virus particles get attached to the host cell through the surface receptors. The nucleocapsid containing relaxed circular DNA (RC DNA) is released into cytoplasm and is transported into the nucleus, where it is repaired to form covalently closed circular DNA (cccDNA). The cccDNA is then transcribed to form subgenomic RNA (sgRNA) and pregenomic RNA (pgRNA). This pgRNA is encapsidated together with P protein and is reversely transcribed to form negative strand DNA. The plus strand synthesis from negative strand results in the generation of new RC DNA. The nucleocapsid containing RC DNA can be used for intracellular cccDNA amplification or they are enveloped and released as virions [12-14].

## Features of HBx Gene

HBx gene is the smallest of all the four partially overlapping ORFs of HBV. It encodes a polypeptide of 154 amino acids with a molecular weight of 17 kDa. It is commonly present in the cytoplasm and to a lesser extent in the nucleus of hepatocytes. The three-dimensional structure of HBx is not much known due to the lack of results from X-ray crystallography and nuclear magnetic resonance studies. Among different species of mammalian *Hepadnavirus*, the comparative analyses of HBx gene sequences showed areas of high conservation like potential coiled-coil motif and presumptive helical domains in amino and carboxy terminal region [15, 16]. There is also some evidence for the linkage of cysteine residues in the N terminus of HBx gene/protein with cysteine residues in the C terminus through disulfide bonds and the phosphorylation of HBx when expressed in insect cells and Hep G2 cells (a human hepatoblastoma cell line) [17].

Even though functions of HBx protein are not fully understood, it is known to have a key role in the development of HCC [17].

## Association Between HBx Gene and p53 and Role in Apoptosis

Tumor suppressor genes, also called anti-oncogenes are a class of genes that are normally involved in regulating cell growth but that may become cancer-causing when damaged. More than 50% of human hepatocarcinoma cases showed mutation of p53 gene, which is located in the short arm of chromosome 17 [18].

Another mechanism by which HBx can mediate HCC is through its effects on apoptosis. HBx protein is known to have both pro-apoptotic and anti-apoptotic effects. The anti-apoptotic activity of HBx protein may be induced by its association with p53, the tumor suppressor gene by inhibiting p53 mediated apoptosis. The inhibition may be due to the failure of action of p53 to upregulate genes such as p21 WAFI, Bax or Fas in presence of HBx. These are the genes which are involved in apoptosis and are upregulated during p53 mediated apoptosis. HBx protein can form a complex with p53 by binding to its C-terminus region and thus sequesters p53 in the cytoplasm and preventing it from entering into the nucleus and inactivating p53 mediated activities like apoptosis and blocks transcriptional transactivation [19, 20].

It is considered that HBx antigen (HBx Ag) may be a protein kinase having auto phosphorylating activity and the phosphorylation of HBx Ag in human hepatoma cells may contribute to HCC. Although the targets for phosphorylation of HBx Ag are not known, phosphorylation of tumor suppressor gene products and cell cycle proteins are recognized to alter their activities in controlling cell growth [21, 22].

## Role of HBx in Transcriptional Transactivation

The studies on HBx transgenic mice indicated evidences of hepatocarcinogenic effects of the gene. HBx transgenic mice showed the characteristics of malignant carcinoma through a series of changes in liver beginning with pre-neoplastic lesions, then to benign adenomas and finally the development of HCC [23, 24]. Not all mice developed HCC and reports indicate that the HBx expression above certain threshold level may cause the transformation to HCC [25]. HBx can act as a transcriptional transactivator that can upregulate the expression of certain proto oncogenes like c-Myc, and c-Jun, transcriptional factors like NF-kB, AP-1, AP-2, TATA binding protein and ATF/cAMP response element binding protein and also other viral genes like enhancers in the nucleus. Among these HBx can directly or indirectly affect NF-kB and AP-1 activity which may lead to acceleration of cell cycle progression, increased proliferation and finally may contribute to control of apoptosis [26, 27]. HBx can function as a transcriptional activator through its interaction with nuclear transcription factors and controlling of cytoplasmic signal transduction pathways like Ras, Raf, mitogen activated protein kinase, Janus family tyrosine kinases-signal transducer and activators of transcription, focal adhesion kinase and protein kinase cascade pathways [28, 29]. The transcriptional activation has a major role in the replication of the virus and the activation of signaling pathways may cause the transactivation of signaling cascades and oncogenes capable of proliferation of hepatocyteswhich may lead to HCC [30].

## HBx in Regulation of Cell Signaling Pathways

There are certain signal transduction pathways that appear to be affected by HBx like stress-activated protein kinases/Jun amino-terminal kinases, extracellular signal regulated kinases, PKB/Akt, Ras-Raf-mitogen activated protein kinase, signal transducer and activators of transcription and Janus family tyrosine kinases and focal adhesion kinase. HBx can activate this cell signaling pathway but the role of HBx on this effect is not defined clearly, but it is assumed that they have a role in hepatocyte proliferation, apoptosis and regulation of cell growth control [31, 32].

## Role of HBx in Nucleotide Excision Repair (NER)

The interaction of HBx with p53 causes the inhibition of cellular functions such as NER. HBx binding with p53 inhibits the interaction between p53 and DNA repair proteins XPB and XPD which are part of TFIIH transcription-NER complex that are involved in inducing apoptosis. This in turn will lead to the disruption of p53 mediated apoptosis and inhibits p53 dependent DNA repair efficiency [33]. Thus the alteration of DNA repair mechanism which can be mediated directly or indirectly through the inhibition of p53 may contribute to carcinogenesis [34].

## Conclusion

The evidences from various experimental studies revealed HBV is associated with HCC by its presence in the tumor cell and also the role of HBV infection in the development of HCC. Although many studies assume HBx gene plays a crucial role in developing HBV infection to HCC, the exact role of HBx gene and its influence in HBV mediated HCC are still unclear. Thus future researches will aim in development of new strategies that help in improved means of early detection and treatment of HCC.
